# Cupping therapy versus acupuncture for pain-related conditions: a systematic review of randomized controlled trials and trial sequential analysis

**DOI:** 10.1186/s13020-017-0142-0

**Published:** 2017-07-24

**Authors:** Ya-Jing Zhang, Hui-Juan Cao, Xin-Lin Li, Xiao-Ying Yang, Bao-Yong Lai, Guo-Yang Yang, Jian-Ping Liu

**Affiliations:** 1 Centre for Evidence-Based Chinese Medicine, Beijing University of Chinese Medicine, 11 Bei San Huan Dong Lu, Beijing, 100029 China; 2 The National Research Center in Complementary and Alternative Medicine (NAFKAM) Department of Community Medicine, Faculty of Health Science, UiT, The Arctic University of Norway, 9037 Tromsø, Norway; 3 National Institute of Complementary Medicine, Western Sydney University, Sydney, NSW 2751 Australia

**Keywords:** Acupuncture, Cupping therapy, Randomized controlled trial, Systematic review, Trial sequential analysis

## Abstract

**Background:**

Both cupping therapy and acupuncture have been used in China for a long time, and their target indications are pain-related conditions. There is no systematic review comparing the effectiveness of these two therapies.

**Objectives:**

To compare the beneficial effectiveness and safety between cupping therapy and acupuncture for pain-related conditions to provide evidence for clinical practice.

**Methods:**

Protocol of this review was registered in PROSPERO (CRD42016050986). We conducted literature search from six electronic databases until 31st March 2017. We included randomized trials comparing cupping therapy with acupuncture on pain-related conditions. Methodological quality of the included studies was evaluated by risk of bias tool. Mean difference, risk ratio, risk difference and their 95% confidence interval were used to report the estimate effect of the pooled results through meta-analysis or the results from each individual study. Trial sequential analysis (TSA) was applied to adjust random errors and calculate the sample size.

**Results:**

Twenty-three randomized trials with 2845 participants were included covering 12 pain-related conditions. All included studies were of poor methodological quality. Three meta-analyses were conducted, which showed similar clinical beneficial effects of cupping therapy and acupuncture for the rate of symptom improvement in cervical spondylosis (RR 1.13, 95% CI 1.01 to 1.26; n = 646), lateral femoral cutaneous neuritis (RR 1.10, 95% CI 1.00 to 1.22; n = 102) and scapulohumeral periarthritis (RR 1.31, 95% CI 1.15 to 1.51; n = 208). Results from other outcomes (such as visual analogue and numerical rating scale) in each study also showed no statistical significant difference between these two therapies for all included pain-related conditions. The results of TSA for cervical spondylosis demonstrated that the current available data have not reached a powerful conclusion. No serious adverse events related to cupping therapy or acupuncture was found in included studies.

**Conclusion:**

Cupping therapy and acupuncture are potentially safe, and they have similar effectiveness in relieving pain. However, further rigorous studies investigating relevant pain-related conditions are warranted to establish comparative effectiveness analysis between these two therapies. Cost-effectiveness studies should be considered in the future studies to establish evidence for decision-making in clinical practice.

## Background

Traditional Chinese non-pharmaceutical therapies, such as acupuncture, are applied under the guidance of the Traditional Chinese Medicine (TCM) theory of syndrome differentiation. As an important part of TCM, these therapies mainly use manual or technique stimulations at specific body parts (especially acupoints) to dredge the meridian system. Generally, non-pharmaceutical therapies, including acupuncture, cupping therapy, moxibustion, massage (*tuina*), and *guasha*, are more likely to be accepted by patients since they have been used in treating numerous diseases or conditions and may have fewer side effects than drugs [1]. Acupuncture, as one of the most popular non-pharmaceutical therapy, has been widely used to treat diseases by regulating the functions of *qi* (vital energy) and blood of the organs through puncturing certain acupoints of meridians in the body with needles, to strengthen the resistance of the body against diseases [2]. A current clinical guideline issued by the American College of Physicians (ACP) recommends non-pharmacologic treatments (such as acupuncture, massage and superficial heat) as the priority treatments to patients with acute, subacute or chronic low back pain [3]. In addition, systematic reviews have reported that acupuncture was indicated for the treatment of chronic pain, mainly headaches, migraines, cervical pain, back pain, and pain from osteoarthritis [4–9].

Cupping therapy also belongs to TCM non-pharmaceutical therapy, which has been used for long time [10]. Cupping practitioners utilize the flaming heating power to achieve suction (minus pressure) inside the glass cups to make them apply on the desired part of the body, and this suction on selected acupoints produces hyperemia or hemostasis, which may result in a therapeutic effect [11]. There are different types of cupping including retained cupping, flash cupping, moving cupping, wet cupping, medicinal cupping, and needling cupping [12]. Since a report about the Olympic swimmer Michael Phelps using cupping therapy to relieve his muscular discomfort, this treatment has become more and more popular outside China. However, although beneficial effects of cupping therapy have been reported in treating various diseases/conditions, there is lack of high-quality evidence to confirm its efficacy [13]. Our previous systematic reviews on cupping for pain-related conditions also identified no high-quality evidence to prove its effectiveness [14, 15].

Both acupuncture and cupping therapy are commonly used in treating similar conditions, especially pain-related conditions. Though the mechanism of acupuncture and cupping therapy may be different, both, therapies employ the meridian and acupoints to activate blood stasis and regulate the flow of *qi* to relieve pain. Cupping therapy has more advantages than acupuncture, such as a non-invasive therapy with relatively shorter treatment duration and potential less treatment cost. It is worthy to critically review the evidence of the comparison of these two therapies to inform clinical practice. Herein, to the objective of this review is to comprehensively review the evidence from randomized controlled trials (RCTs) comparing cupping therapy with acupuncture for pain-related conditions.

## Methods

The protocol of this review was registered in PROSPERO (CRD42016050986) on 15th November 2016 (Achieved at http://www.crd.york.ac.uk/PROSPERO/). Since pain-related conditions were most commonly treated by cupping therapy and acupuncture, we limited the target conditions (such as musculoskeletal pain, tissue pain and neuralgia pain) in this review to reduce the clinical heterogeneity among included studies.

### Inclusion criteria

RCTs comparing cupping therapy with acupuncture were included. Pain-related conditions were classified by the type of tissue according to international statistical classification of disease and health related problems by World Health Organization [16], including musculoskeletal system pain (such as spondylopathies, lumbar spondylosis, knee osteoarthritis, acute tissue pain), and neurologic pain (such as lateral femoral cutaneous nerve and herpes zoster pain). Acupuncture is defined as the insertion of fine needles, sometimes in conjunction with electrical stimulus, to influence physiological functioning of the body. In this review, we included both manual acupuncture (including auricular therapy, scalp needle, and abdominal acupuncture) and electro-acupuncture. Cupping is defined that practitioners utilize the flaming heating power to achieve suction (minus pressure) inside the glass cups to make them apply on the desired part of the body. In this review, all types of cupping (i.e. wet cupping, herbal cupping, moving cupping, flash cupping or retained cupping) were included. Primary outcome measures included severity of pain, functional capacity, quality of life (QoL). Secondary outcomes included depression, rate of symptom improvement and adverse effects. There was no limitation on language and publication type.

### Identification and selection of studies

We searched China Network Knowledge Infrastructure (CNKI), Chinese Scientific Journal Database (VIP), Wan Fang Database, PubMed, EMBASE, and the Cochrane Library, all the searches ended at March 2017. The search terms included acupuncture-related terms (i.e. “acupuncture”, “acupoint”, “needle”, “electroacupuncture”, “manual acupuncture”, “auricular needling”, “scalp needle”, or “abdominal acupuncture”), combined with cupping-related terms (i.e. “cupping therapy”, “bleeding cupping”, “wet cupping”, “dry cupping”, “flash cupping”, “herbal cupping”, “moving cupping” or “retained cupping”) and pain-related terms (i.e. “ache”, “pain”, “painful”, or “analgesic”). Two authors (XY Yang and BY Lai) selected studies for eligibility and checked against the inclusion criteria independently.

### Data extraction and risk of bias assessment

Two authors (YJ Zhang and XL Li) extracted the data from the included studies independently. The methodological quality of RCTs was assessed according to the criteria from the Cochrane Handbook for Systematic Reviews of Interventions [17]. The quality of studies was categorized into low, unclear, or high risk of bias according to the risk for each important outcome within included studies, including adequacy of generation of the allocation sequence, allocation concealment, blinding, whether there were incomplete outcome data and selected reporting the results. Studies which met all criteria were categorized to low risk of bias, studies which meet none of the criteria were categorized to high risk of bias, and those were categorized to unclear risk of bias if insufficient information acquired to make the judgment.

### Data analysis

Data were extracted and calculated for frequency using Microsoft Excel 2007 (American: The Microsoft Corporation, 2007). Binary outcomes were summarized using risk ratio (RR) with 95% confidence intervals (CI) for relative effect and risk difference (RD) with 95% CI for absolute effect. The continuous outcomes were summarized using mean difference (MD) with 95% CI. Revman 5.3 (Copenhagen: The Nordic Cochrane Centre, The Cochrane Collaboration, 2016) was used for data analyses. Meta-analysis was used if the studies had similar clinical characteristics (such as study design, participants, interventions, control, and outcome measures) and acceptable statistical heterogeneity. Random-effect model was used for meta-analysis. Statistical heterogeneity was detected by *I* ^2^ test, an *I* ^2^ > 50% indicates the possibility of statistical heterogeneity among the studies. If *I* ^2^ was larger than 75%, which means there was obviously statistical heterogeneity among studies, only results from each single study were present respectively rather than pooling analysis. Funnel plot analysis was planned to be generated to detect publication bias.

### Trial sequential analysis (TSA)

TSA can be performed if there are more than 5 included studies in the meta-analysis. We applied TSA version 0.9.5.5 (Copenhagen: The Copenhagen Trial Unit, Center for Clinical Intervention Research, 2016) to calculate the required sample size in a meta-analysis and to detect the robustness of the result. We used the diversity-adjusted required information size estimated from a control event proportion of the included studies and a priori intervention effect of 5%, and the diversity which was estimated in the included studies.

## Results

### Basic information of included studies

**Fig. 1:**
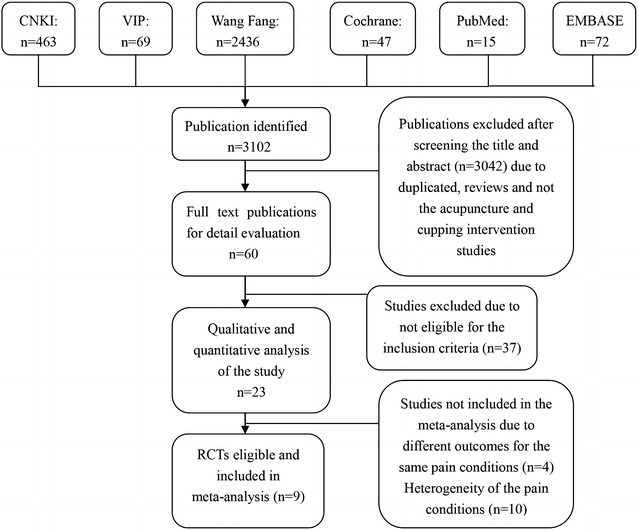
Flow chart of the study

After primary searches from the 6 databases, 3102 studies were identified. After screening title and abstract, the majority of studies were not included due to obvious ineligibility, and full texts of 3079 studies were retrieved. Finally, 23 RCTs [18–40] were included in this review, 21 of which were published in Chinese and the remaining 2 [21, 40] were published in English. All of the 23 studies were conducted in China (see Fig. 1).

### Description of interventions

All the included studies compared cupping therapy with acupuncture. In the cupping group, 20 (86.96%) studies used wet cupping, and 3 [33, 36, 40] (13.04%) studies used at least two types of cupping methods. Treatment frequency in 3 [33, 36, 37] (13.04%) studies was once daily, in 8 [19, 25, 27, 29, 31, 32, 38, 39] (34.78%) studies was once every 2 days, in 1 [21] (4%) study was twice weekly, in 6 [18, 20, 22–24, 40] (26.08%) studies was 3 times weekly and in other 2 [34, 35] studies (8.6%) was once weekly. The remaining 3 [26, 28, 30] (13.04%) studies not reported the frequency of the treatment. The treatment duration per session in 22 (95.7%) studies was 3–10 min, and in the remaining 1 [27] study was 30 min. In the acupuncture group, 22 (95.6%) studies used manual acupuncture, and 1 (4.3%) study used electroacupuncture. Treatment frequency in 13 [18–20, 22–24, 29, 31, 32, 34–36, 38] (56.52%) studies was once a day, in 4 [27, 28, 30, 39] (17.39%) studies was once 2 days, in 1 [21] (4.3%) study was once weekly, in another 1 [40] (4.3%) study was 3 times weekly. The remaining 4 (17.39%) studies not reported the treatment frequency. The treatment duration per session was 20–30 min (Table 1).

**Table 1:** Characteristics of 23 included trials comparing cupping therapy and acupuncture for pain-related conditions

Study ID	Diseases	Sample size	Age	Gender	Cupping group	Acupuncture group	Treatment duration (days)	Outcomes
		(Rx/C)	Year (Rx/C)	M/F				
Dong [18]	Cervical spondylosis of vertebral artery type	60/60	40.62 ± 14.57 41.15 ± 13.34	33/27 34/26	Wet cupping on Haoyi acupoint. If not effect, wet cupping on Zhengumai acupoint after 3 days and evaluate the effect on the seventh day	Acupuncture on bilateral cervical (C3–7), Jiaji (EX-B2) points, Dazhui (DU14), Fengchi (GB20), Neiguan (PC6), Hegu (LI4) with stimulation. 1×/day	15	Symptom score, total effective rate
Zhou [19]	Cervical spondylosis	100/100	20–60	Not reported	With Zhuang medicine lotus needle pricking and cupping therapy. 1×/2 days	Acupuncture on bilateral cervical (C3–7), Jiaji (EX-B2) points, Dazhui (DU14), Fengchi (GB20), Neiguan (PC6), Hegu (LI4) with stimulation. 1×/day	20	VAS, symptom improvement rate
Hu [20]	Cervical spondylosis of vertebral artery type	20/20	40.9 ± 3.5 42.8 ± 1.6	12/8 10/10	Wet cupping on Haoyi acupoint. If not effect, wet cupping on Zhengumai acupoint after 3 days and evaluate the effect on the seventh day	Acupuncture on bilateral cervical (C3–7), Jiaji (EX-B2) points, Dazhui (DU14), Fengchi (GB20), Neiguan (PC6), Hegu (LI4) with stimulation 30 min. 1×/day	15	Symptom improvement rate
Mou [21]	Cervical spondylotic radiculopathy	68/56	46.4 ± 11.6 47.8 ± 11.9	34/34 24/32	Wet cupping on Dazhui (GV14) and Jianjing (GB21) acupoint 2×/week	Acupuncture on bilateral cervical (C3–7), Jiaji (EX-B2) points and Jianjing (GB 21) on the affected side, 30 min, 2×/week	60	VAS, NDI, CAS
A [22]	Cervical spondylosis	43/43	45.3 ± 9.4 44.2 ± 9.2	23/20 22/21	Wet cupping on Haoyi acupoint. If not effect, wet cupping on Zhengumai acupoint after 9 days	Acupuncture on bilateral cervical (C3–7), Jiaji (EX-B2) points, Dazhui (DU14), Fengchi (GB20), Neiguan (PC6), Hegu (LI4) with stimulation, 1×/day, 25 min	14	Symptom improvement rate
Wang [23]	Cervical spondylosis	72/72	45.9 ± 8.7 45.1 ± 7.9	79/65	Wet cupping on Haoyi acupoint for 10 min. If not effect, wet cupping on Zhengumai acupoint after 3 days	Acupuncture on Fengchi (GB20), Wangu (GB12), Sishencong (EX-HN1), Baihui (GV20), Tianzhu (BL10), Yunting, Jiaji (EX-B2), 1×/day, 25 min	14	Symptom improvement rate
Na [24]	Cervical spondylosis	28/28	45.1 ± 8.5 44.3 ± 8.9	15/13 17/11	Wet cupping on Haoyi acupoint. If not effect, wet cupping on Zhengumai acupoint after 9 days and evaluate the effect on the 12 day	Acupuncture on bilateral cervical (C3–7), Jiaji (EX-B2) points, Dazhui (DU14), Fengchi (GB20), Neiguan (PC6), Hegu (LI4) with stimulation, 1×/day, 25 min	14	Symptom improvement rate
Wang [25]	Knee osteoarthritis	40/40	42–68	62/58	Wet cupping on tender point (Ashi), Xiyan (EX-LE5), Heding (EX-LE2), Yanglingwquan (GB34), Liangqiu (ST34), 1×/2 days	Acupuncture: abdominal acupuncture on Zhongwan (N12), Guanyuan (N4), Shuifen (RN9), Qipang, Waling, Xiafengshidian, Xiafenshineidian with stimulation, 30 min	23	Symptom improvement rate
Wang [26]	Knee osteoarthritis	89/82	59 ± 9 61 ± 8	18/71 21/61	Wet cupping on Xiyan (EX-LE2), tender acupoints (Ashi) for 5–10 times	Acupuncture on Xiyan (EX-LE2), tender acupoints (Ashi), 20 min for 5–10 times	28	VAS, WOMAC
Shu [27]	Lateral femoral cutaneous neuritis	25/25	50.32 ± 10.44 50.88 ± 9.27	14/11 12/13	Wet cupping on tender point and Yanglingquan (GB34), 30 min, 1×/2 days	Local multiple superficial needling, 30 min, 1×/2 days	21	Symptom improvement rate
Zhang [28]	Lateral femoral cutaneous neuritis	26/26	46.9 47.6	20/6 21/5	Wet cupping on tender point	Local Multiple superficial needling, 30 min, 1×/2 days	21	Symptom improvement rate
Wang [29]	Lumber disk herniation	32/30	20–60	48/45	Wet cupping in tender point (Ashi) and surface reflect lesion site and retained for 5–1 min, 1×/2 days	Acupuncture on traditional site, 30 min, 1×/day.	28	JOA score, symptom improvement rate
Zhou [30]	The third lumber vertebral transverse process syndrome	60/60	35.1 35.9	38/22 34/26	Wet cupping on tender point (Ashi) and retained cupping for 5 min	Acupuncture on Ashi, Jiaji (EX-B2), Huantiao (GB30), Weizhong (BL40), 20 min, 1×/2 days	28	VAS, Symptom improvement rate
Zhang [31]	Postherpetic neuralgia	20/20	61 ± 7 60 ± 7	12/8 13/7	Wet cupping on Ashi for 3–10 min, 1×/2 days	Acuponcture around the pain site for 20–30 mins, 1×/day	10	Symptom improvement rate
Huang [32]	Postherpetic neuralgia	49/47	65.35 64.28	28/21 25/22	Wet cupping with Zhuang lotus needle on Longji, Jiaji (EX-B2), Jianjing (GB21), Ashi and retained cupping for 5–10 min, 1×/2 days	Acupuncture on Quchi (LI11), Hegu (LI4), Taichong (LR3), Sanyinjiao (SP6), Xuehai (SP10), Zusanli (ST36) and tender points (Ashi), 25 min. 1×/day	30	Symptom improvement rate, VAS
Wu [33]	Toothache	204/203	Not reported	Not reported	Wet cupping on Dazhui (DU14), Jiaji (EX-B2) and flash cupping around Dazhui for 10–15 min, 1×/day	Acupuncture on traditional site	Not Reported	Symptom improvement rate
Bao [34]	Scapulohumeral periarthritis	52/52	57.1 ± 7.9 56.2 ± 8.6	28/24 26/26	Wet cupping on Jian, Jianqian, Jianhou and Ashi, 1×/week	Acupuncture on Jinayu (LI15), Jianzhen (SI9), Jianliao (SJ14), Jinaqian, Quchi (LI11), Waiguan (SJ5), Yanglingquan (GB34), 30 min, 1×/day	15	Symptom improvement rate
Sha [35]	Scapulohumeral periarthritis	52/52	51.1 ± 5.8 51.3 ± 6.5	28/24 29/23	Wet cupping on Jian, Jianqian, Jianhou and Ashi, 1×/week	Acupuncture on Jinayu (LI15), Jianzhen (SI9), Jianliao (SJ14), Jinaqian, Quchi (LI11), Waiguan (SJ5), Yanglingquan (GB34), 30 min, 1×/day	15	Symptom improvement rate
Liu [36]	Muscles fibrositis	38/38	30–60	19/19 23/15	Flash cupping on tender points and retained for 10–15 min, 1×/day	Electricacupuncture on traditional acupoints. 1×/day	16	Symptom improvement rate
Huang [37]	Soft tissue contusion	132/132	4–69	107/157	Wet cupping on Ashi. 1×/day	Resistance acupuncture on the contusion site	7	Symptom improvement rate
Zhou [38]	Acute lumbar sprain	26/26	41.27 ± 8.76 39.38 ± 8.12	9/17 12/14	Wet cupping on waist area of the bladder meridian for 5 mins, 1×/2 days	Acupuncture on bladder meridian for 30 min, 1×/day	10	Symptom improvement rate, temperature difference of body surface
Wang [39]	Acute ankle joint	47/73	9–60	36/37 25/22	Wet cupping on Ashi, 10 min 1×/2 days	Acupuncture on Ashi or surrounding acupoint, 1×/2 days	20	Symptom improvement rate
Cao [40]	Fibromyalgia	29/27	Not reported	Not reported	Cupping for tender points, 3×/week	Acupuncture for tender points, 3×/week	35	VAS, SF36, HAMD

*VAS* Visual Analogue Scale, *NDI* neck disability index (physical therapy), *CAS* the Clinical Assessment Scale, *WOMAC* the Western Ontario and McMaster Universities osteoarthritis index, *JOA* the Joint Operational Area, *SF-36* the MOS item short from health survey, *HAMD* the Hamilton Depression Scale

### Description of acupoints

In the cupping group, 22 (95.7%) studies used wet cupping on tender points (Ashi), and 1 [38] (4.3%) studies chose the acupoints on the meridians passing the pain area. In the acupuncture group, 4 [31, 37, 39, 40] (17.3%) studies only used tender points (Ashi) the remaining 19 (82.6%) studies chose the acupoints on the meridian of pain and acupoints according to syndrome differentiation.

In general, the number of acupoints in acupuncture group is more than that in cupping therapy. According to the included studies of this review, it’s interesting to find that acupuncturists often selected acupoints according to syndrome differentiation, as well as the tender points (Ashi), while cupping therapy practitioners were more likely to choose the tender points (Ashi) for retaining cupping.

### Distribution of diseases/conditions

Twelve diseases or conditions were treated in the included RCTs (Table 1). The top 6 diseases/conditions are cervical spondylosis [18–24], knee osteoarthritis [20, 21], lateral femoral cutaneous neuritis [27, 28], lumber disk herniation [29, 30], postherpetic neuralgia [31, 32] and scapulohumeral periarthritis [34, 35].

The pain-related conditions included locomotor pain (cervical spondylosis, lumber disk herniation, scapulohumeral periarthritis and knee osteoarthritis); tissue injury (soft tissue contusion, acute ankle joint); muscle pain (muscles fibrositis and fibromyalgia), neuralgia pain (lateral femoral cutaneous neuritis and postherpetic neuralgia) and acute pain (toothache). Relieving pain was the main goal of the treatments for the included trials, and the study purposes of them were to compare the effectiveness on pain reduction between acupuncture and cupping therapy.

### Outcome assessment

Twenty studies (86.95%) used the rate of symptom improvement as the primary outcome, which was defined as the rate of the sum of the cured, markedly effective and effective improvement. Symptom improvement was categorized into four grades (i.e. cured, markedly effective, effective, and ineffective) according to the changes or improvement of the symptoms. ‘The cured’ was the disappearance of the pain symptoms, ‘markedly effective’ was the almost disappearance of the symptoms, ‘effective’ was symptoms alleviation, and ‘ineffective’ was no changes in symptoms. Besides, 8 [18, 19, 21, 26, 29, 30, 32, 40] studies (34.78%) used numerical scales to evaluate the severity of the symptoms, including the Visual Analogue Scale (VAS) for pain, the neck disability index (NDI), the Clinical Assessment Scale (CAS), the Western Ontario and McMaster Universities osteoarthritis index (WOMAC), the Joint Operational Area (JOA) Scores for joint mobility, the MOS item short from health survey (SF-36), and the Hamilton Depression Scale (HAMD) for social activity.

### Risk of bias assessment

**Fig. 2:**
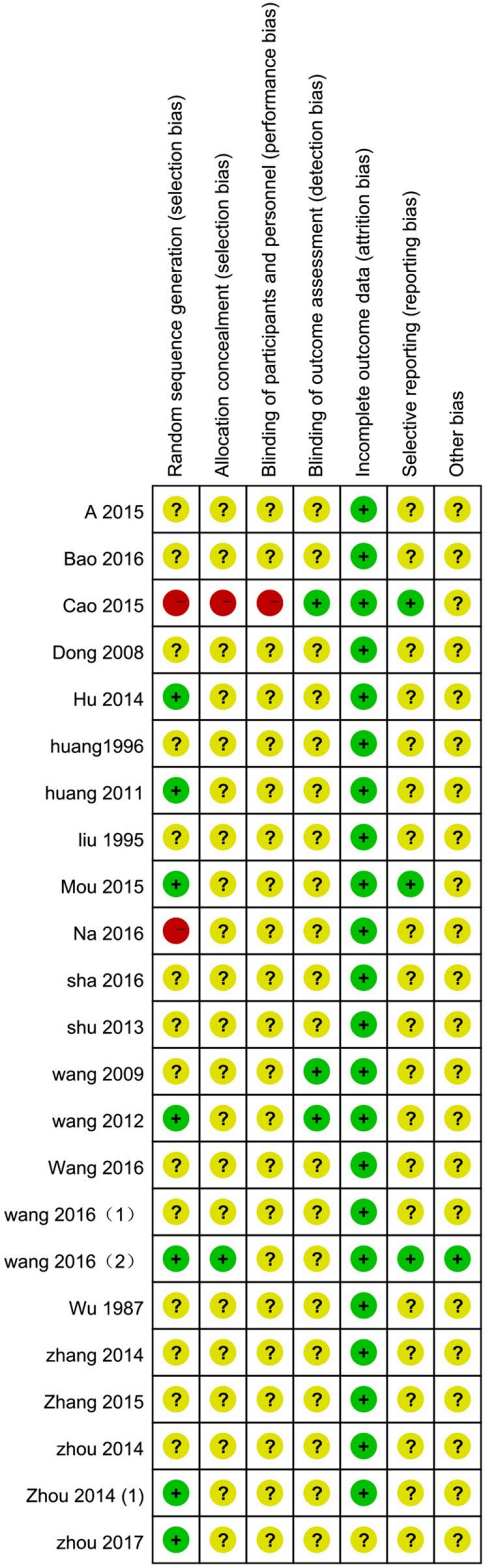
Risk of bias summary

According to our pre-defined methodological quality criteria, no study was evaluated as low risk of bias, 8 [19–21, 26, 29, 31, 32, 40] studies unclear risk of bias, and the remaining 15 studies high risk of bias. The sample size in each study varied from 40 to 407 participants with an average of 56 patients per group. None of the studies specified the sample size calculation. Eleven studies described the details of randomization procedure. Among them, 6 studies [19–21, 26, 29, 32] used a random number table to generate the random allocation. One study [27] was declared as ‘randomized controlled trial’, however, it used a quasi-randomization method by dividing participants into two groups based on the sequence of the registration order. Only 1 [26] of the above 11 studies reported using sealed envelope to implement allocation concealment. Three studies [31, 39, 40] mentioned they used blinding method, in which 2 studies [31, 40] reported that they blinded outcome assessors, while the other one did not specify who were blinded. Two studies [21, 26] reported the number of dropouts, but none of them used intention-to-treat analysis or other appropriate methods to deal with the missing data (Fig. 2).

There were 19 (82.6%) studies reported the comparability of baseline, but only 1 [40] study reported the baseline data of both the outcomes and the demographic characteristics of participants. Nine [19, 21, 26, 29, 31, 32, 34, 39, 40] (39.13%) studies reported the inclusion criteria, and 9 [18, 19, 21, 26, 29, 31, 32, 38, 40] (39.13%) studies reported the exclusion criteria. Seventeen (73.91%) studies reported the diagnostic criteria (as we mentioned before). Only one protocol of a study [40] was available which was registered in Clinicaltrials.gov, thus we acquired the protocol to supporting the judgment of reporting bias of this study and it was of low risk.

### Effect estimates

#### Results from individual trials

Due to the variations in study quality, participants’ characteristics and outcome measures of the included RCTs, data from 14 studies could not be synthesized by meta-analysis. Among them, 11 [21, 25, 29–33, 36–38, 40] studies (78.5%) reported that there was no statistical significant difference between cupping and acupuncture on the rate of symptom improvement for specific conditions, including cervical spondylotic radiculopathy, lumber disk herniation, knee osteoarthritis, toothache, muscles fibrositis, soft tissue contusion, acute lumber sprain, postherpetic neuralgia and fibromyalgia. Three [19, 26, 39] studies reported cupping might have better effects than acupuncture on increasing the total effective rate in acute ankle joint knee osteoarthritis, acute lumbar sprain and the third lumber vertebral transverse process syndrome according to the VAS scores. Table 2 shows the detailed results of the 14 studies.

**Table 2:** Estimate effect of cupping therapy and acupuncture for pain-related conditions

Study ID	Sample size	Disease	Estimate effect	P	Outcomes
Zhou [19]	200	Cervical spondylosis	MD −1.70, 95% CI −1.94 to −1.46	P < 0.00,001	VAS for pain
Mou [21]	124	Cervical spondylotic radiculopathy	MD 0.11, 95% CI −0.78 to 1.00 MD 4.33, 95% CI −3.14 to 11.80 MD 1.59, 95% CI −1.17 to 4.35	P = 0.81 P = 0.26 P = 0.26	VAS Neck disability index (physical therapy) CAS
Wang [25]	80	Knee osteoarthritis	RR 1.29, 95% CI 0.32 to 5.19 RD 0.03, 95% CI −0.11 to 0.16	P = 0.72 P = 0.72	Symptom improvement rate
Wang [26]	171	Knee osteoarthritis	MD −0.97, 95% CI −1.56 to −0.38 MD −1.35, 95% CI −1.91 to −0.79 MD −1.01, 95% CI −1.87 to −0.15 MD −0.17, 95% CI −0.52 to 0.18 MD −2.92, 95% CI −6.18 to 0,34 MD −4.14, 95% CI −8.49 to 0.21 MD −1.66, 95% CI −2.48 to −0.84 MD −0.35, 95% CI −0.68 to −0.02 MD −4.93, 95% CI −7.97 to −1.89 MD −6.93, 95% CI −11.0 to −12.85	P = 0.001 P < 0.00001 P = 0.02 P = 0.34 P = 0.08 P = 0.06 P < 0.0001 P = 0.04 P = 0.001 P = 0.0009	VAS VAS (follow at 4 weeks) WOMAC—pain scores WOMAC—stiff scores WOMAC—difficult of life scores WOMAC —total scores WOMAC—pain scores (follow at 4 weeks) WOMAC—stiff scores (follow at 4 weeks) WOMAC—difficult of life scores (follow at 4 weeks) WOMAC total scores (follow at 4 weeks)
Wang [29]	62	Lumber disk herniation	MD 1.88, 95% CI −0.24 to 4.00 RR 0.89, 95% CI 0.63 to 1.27 RD −0.07, 95% CI −0.31 to 0.16	P = 0.08 P = 0.53 P = 0.53	JOA scores Symptom improvement rate
Zhou [30]	120	The third lumber vertebral transverse process syndrome	RR 1.05, 95% CI 0.96 to 1.15 RD 0.05, 95% CI −0.03 to 0.13 MD −1.58,95% CI −2.04 to −1.12	P = 0.25 P = 0.24 P < 0.00001	Symptom improvement rate VAS scores
Zhang [31]	40	Postherpetic neuralgia	RR 0.95, 95% CI 0.83 to 1.09 RD −0.05, 95% CI −0.18 to 0.08 MD 0.10, 95% CI −0.51 to 0.70	P = 0.47 P = 0.44 P = 0.75	Symptom improvement rate VAS
Huang [32]	96	Postherpetic neuralgia	RR 1.16, 95% CI 1.00 to 1.33 RD 0.13, 95% CI 0.01 to 0.25 MD −1.17, 95% CI −1.67 to −0.67	P = 0.05 P = 0.04 P < 0.00001	Symptom improvement rate VAS
Wu [33]	407	Toothache	RR 1.04, 95% CI 1.00 to 1.08 RD 0.03, 95% CI 0.00 to 0.07	P = 0.03 P = 0.03	Symptom improvement rate
Liu [36]	76	Muscles fibrositis	RR 1.05, 95% CI 0.96 to 1.15 RD 0.05, 95% CI −0.03 to 0.14	P = 0.24 P = 0.22	Symptom improvement rate
Huang [37]	264	Soft tissue contusion	RR 0.99, 95% CI 0.93 to 1.05 RD −0.01, 95% CI −0.06 to 0.05	P = 0.79 P = 0.79	Symptom improvement rate
Zhou [38]	46	Acute lumbar sprain	RR 1.09, 95% CI 0.90 to 1.33 RD 0.08, 95% CI −0.10 to 0.25	P = 0.39 P = 0.38	Symptom improvement rate
Wang [39]	120	Acute ankle joint	RR 1.18, 95% CI 1.04 to 1.33 RD 0.15, 95% CI 0.04 to 0.25	P = 0.009 P = 0.005	Symptom improvement rate
Cao [40]	56	Fibromyalgia	MD −4.06, 95% CI −5.4 to 13.72 MD −5.93, 95% CI −7.89 to 19.75 MD −0.42, 95% CI −4.09 to 4.93		VAS Quality of life HAMD

*VAS* Visual Analogue Scale, *MD* mean difference, *CAS* the Clinical Assessment Scale, *WOMAC* the Western Ontario and McMaster Universities osteoarthritis index, *JOA* the Joint Operational Area, *SF-36* the MOS item short from health survey, *HAMD* the Hamilton Depression Scale

#### Results from pooling analyses

**Fig. 3:**
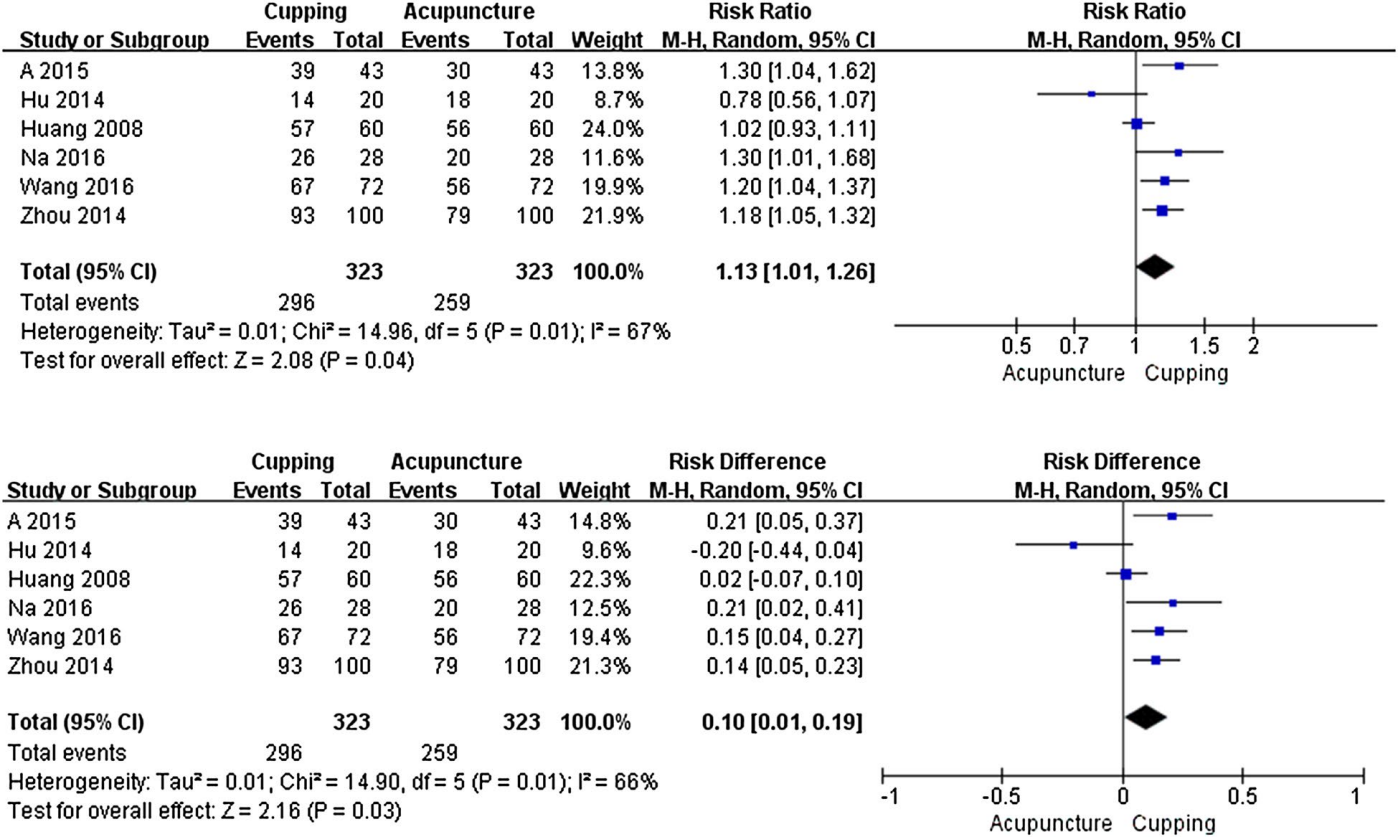
Forest plot. Cupping therapy versus acupuncture for symptom improvement in patients with cervical spondylosis

Seven studies [18–24] compared acupuncture and cupping therapy for cervical spondylosis, 2 [19, 21] of them reported VAS scores and found cupping therapy was superior to acupuncture on pain relief, however, data could not be pooled due to the statistical heterogeneity (*I* ^2^ = 93%). One [18] of these two studies reported that cupping had better effect on reducing VAS scores (MD 1.70 cm, 95% CI 1.46 to 1.94, n = 200) and the other one [20] reported that cupping and acupuncture had similar effects on decreasing VAS scores (MD −0.11 cm, 95% CI −1.0 to 0.78, n = 64). A meta-analysis was conducted with the data from 6 [18–20, 22–24] studies which reported the symptom improvement rate. The result showed that cupping had better effects on increasing symptom improvement rate (RR 1.13, 95% CI 1.01 to 1.26, P = 0.04, *I* ^2^ = 67%; n = 646, 6 trials) than that of acupuncture (Fig. 3). Symptom improvement rate of cupping therapy was 10% higher than acupuncture (RD 0.1, 95% CI 0.01 to 0.19, P = 0.03, *I* ^2^ = 67%; n = 646, 6 trials) (Fig. 3). The remaining 1 study [21] found no significant difference between the two therapies on changing of the VAS (MD −0.11 cm, 95% CI −0.92 to 2.62; n = 124), NDI (MD 4.33, 95% CI −3.14 to 11.80; n = 124), and CAS scores (MD 1.59, 95% CI −1.17 to 4.35; n = 124).

Two studies [27, 28] assessed cupping therapy compared with acupuncture for lateral femoral cutaneous neuritis. The meta-analysis showed similar effects between cupping and acupuncture on increasing symptom improvement rate (RR 1.10, 95% CI 1.00 to 1.22, P = 0.71, n = 100, 2 trials; RD 0.10, 95% CI 0.10 to 0.19, P = 0.71, n = 100, 2 trials).

Two studies [34, 35] assessed cupping therapy compared with acupuncture for scapulohumeral periarthritis. The meta-analysis showed similar effects between cupping and acupuncture on symptom improvement rate (RR 1.31, 95% CI 1.51 to 1.51, P = 0.84; n = 208, 2 trials). Cupping therapy was 22% higher than that of acupuncture (RD 0.22, 95% CI 0.12 to 0.32, P < 0.0001; n = 208, 2 trials) in symptom improvement.

Funnel plot analysis could not be conducted due to the insufficient number of included trials in each meta-analysis.

### Trial sequential analysis

**Fig. 4:**
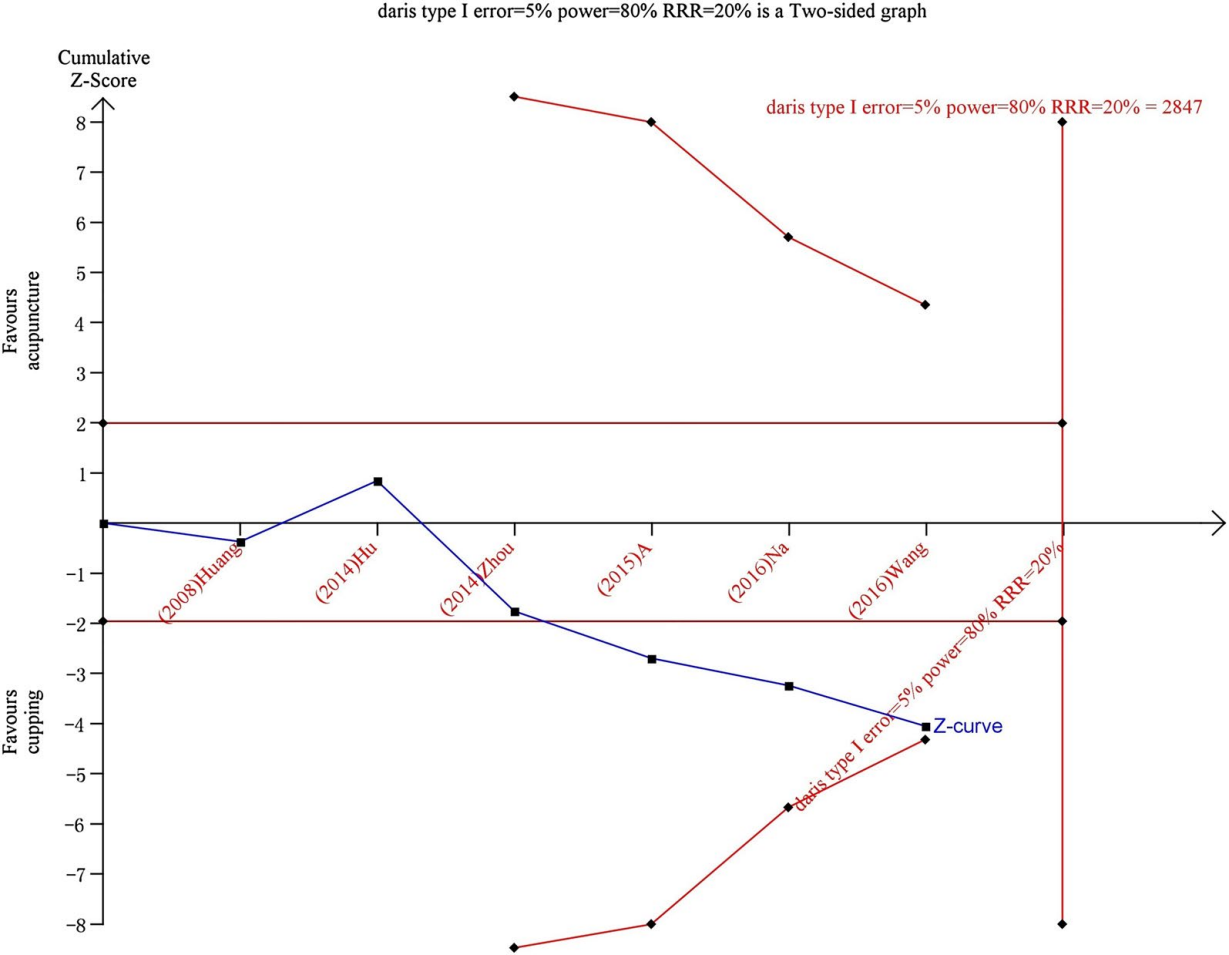
Trial sequential analysis on cupping therapy versus acupuncture for symptom improvement rate in patients with cervical spondylosis

TSA was conducted with the data from 6 [18–20, 22–24] studies which reported the symptom improvement rate for cervical spondylosis. TSA illustrated that the cumulative Z-curve (blue curve) only across the traditional boundary of 5% significance (horizontal red line) but did not cross the monitoring boundaries (red inward sloping curves), which is needed to obtain firm evidence controlling for the risk of random error. This resulted in a required information size of 2847 participants. TSA of all included trials suggests that about 18 high quality RCTs (2201 participants) were required to confirm possible intervention effect (Fig. 4).

### Adverse events

Seventeen (73.92%) out of the 23 studies did not report the outcome on adverse event, and other 6 [24–27, 31, 32] (26.08%) studies reported that there was no adverse event in both groups during the treatment.

## Discussion

### Summary of main findings

Twenty-three RCTs with 12 pain-related conditions were included in this review. The majority of the included studies had poor methodological quality according to cochrane risk of bias assessment. Manual or electro-acupuncture were compared with cupping therapy (including wet cupping, moving cupping, flash cupping and retaining cupping) in these studies. Due to the obvious clinical or statistical heterogeneity, only data from ten studies could be pooled in three meta-analyses for cervical spondylosis, lateral femoral cutaneous neuritis and scapulohumeral periarthritis. It should be point out that though some of the included trials reported statistical significant differences between the two therapies according to the symptom improvement rate or pain relieve assessed by scales (such as VAS), according to clinical experience these differences have not achieved clinically meaningful difference between cupping therapy and acupuncture since the differences were too small (RR less than 2 or MD of the VAS less than 3 cm) [41] and the result of TSA for cervical spondylosis also indicated the character available data has not reached a powerful conclusion. All of included studies showed similar effects of these two therapies on increasing symptom improvement rate and other pain reduction for the covered diseases/conditions. No serious adverse events were reported from both groups.

### Strengths and weakness

To the best of our knowledge, this review is the first systematic review comparing cupping therapy with acupuncture for pain-related conditions. The findings of our review might provide evidence-based information of the therapeutic effects of the two therapies to support clinical treatment decisions on pain-related conditions.

There are some limitations of this review. Only 23 RCTs covering 12 conditions were included, thus for most of the conditions we got only 1 or 2 small studies for each to compare the effect of cupping therapy and acupuncture. Furthermore, the sample size of the included participants was too small to provide a powerful evidence. In addition, only three meta-analyses could be conducted with 2–6 studies in each. Though funnel plot analysis could not be conducted, most of the included studies were conducted and published in China, and outcomes of the original studies all got ‘positive’ conclusions, though some of which did not show any clinically meaningful difference between the two therapies. Therefore, the ‘uncertainty principle’ might be destroyed due to the ‘preference’ of the researchers and lead to publication bias.

### Implication for clinical practice

Regardless the potential bias and limitations of this review, all of the included studies showed that cupping therapy had similar therapeutic effects with acupuncture in treating pain-related conditions (such as cervical pain, back pain, osteoarthritis, and acute soft tissue injury).

For pain-related conditions, cupping therapy and acupuncture have different choice of acupoints. Based on our review, acupuncture often use two basic acupoints which are tenderness point (Ashi) and the meridians passing through the pain area; while for cupping therapy, wet cupping uses special acupoints (such as Haoyi for cervical spondylopathy) for local pain, and flash cupping combined with moving cupping often use the meridians passing through the pain area for widespread pain.

Considering the similar therapeutic effects of these two therapies, clinical practitioners may consider to choose any of them, based on their own clinical experience and the preference of patients.

### Implications for future research

The methodological quality of included studies was general poor, therefore more well designed, conducted and reported RCTs are warranted in the future. According to the Consolidated Standards of Reporting Trials (CONSORT) [42], randomization methods need to be clearly described and fully reported. Although blinding of patients and practitioners might be very difficult for cupping therapy and acupuncture, we strongly suggest the blinding of outcome assessors should be attempted to minimize assessment bias. We also suggest future researchers to select outcome measures based on international consensus for relevant pain conditions. The study protocol is strongly suggested to be registered in authoritative registration plat form [43], such as WHO international Clinical Trial Registration Platform (WHO ICTRP) or Clinicaltrial.com. The reporting of sample size calculation, and using intention-to-treat analysis to deal missing data might be considered.

In addition, due to different treatment durations and variations of the cupping therapy and acupuncture, it is worthy developing cost-effectiveness analyses to further comparing these two TCM non-pharmaceutical therapies, in order to find more economic clinical procedure. Clinical equivalence trials with large sample size will be needed to confirm the non-significant difference of effectiveness between acupuncture and cupping therapy. In addition, compliance, satisfaction and preference of participants to treatment would be considered to employ as outcome measurements in future study.

## Conclusion

Currently, there are limited evidence shows that cupping therapy and acupuncture has similar effect on relieving pain and improving other symptoms of pain-related conditions. Larger and rigorously designed RCTs are needed to confirm this conclusion. Economic studied might be considered in the future to further compare the cost-effectiveness of cupping therapy and acupuncture.

## Abbreviations

CI: confidence intervals
CAS: the Clinical Assessment Scale
HAMD: the Hamilton Depression Scale
JOA: the Joint Operational Area
MD: mean difference
NDI: neck disability index (physical therapy)
RCT: randomized controlled trial
RD: risk difference
RR: risk ratio
REM: random-effect model
SF-36: the MOS item short from health survey
TCM: Traditional Chinese Medicine
TSA: trial sequential analysis
VAS: Visual Analogue Scale
WOMAC: the Western Ontario and McMaster Universities osteoarthritis index
